# Altered Patterns of Amplitude of Low-Frequency Fluctuations and Fractional Amplitude of Low-Frequency Fluctuations Between Amnestic and Vascular Mild Cognitive Impairment: An ALE-Based Comparative Meta-Analysis

**DOI:** 10.3389/fnagi.2021.711023

**Published:** 2021-08-31

**Authors:** Xulian Zhang, Chen Xue, Xuan Cao, Qianqian Yuan, Wenzhang Qi, Wenwen Xu, Shaojun Zhang, Qingling Huang

**Affiliations:** ^1^Department of Radiology, The Affiliated Brain Hospital of Nanjing Medical University, Nanjing, China; ^2^Division of Statistics and Data Science, Department of Mathematical Sciences, University of Cincinnati, Cincinnati, OH, United States; ^3^Department of Neurology, The Affiliated Brain Hospital of Nanjing Medical University, Nanjing, China; ^4^Department of Statistics, University of Florida, Gainesville, FL, United States

**Keywords:** amnestic mild cognitive impairment, vascular mild cognitive impairment, amplitude of low-frequency fluctuations, fractional amplitude of low-frequency fluctuations, resting state

## Abstract

**Background:** Changes in the amplitude of low-frequency fluctuations (ALFF) and the fractional amplitude of low-frequency fluctuations (fALFF) have provided stronger evidence for the pathophysiology of cognitive impairment. Whether the altered patterns of ALFF and fALFF differ in amnestic cognitive impairment (aMCI) and vascular mild cognitive impairment (vMCI) is largely unknown. The purpose of this study was to explore the ALFF/fALFF changes in the two diseases and to further explore whether they contribute to the diagnosis and differentiation of these diseases.

**Methods:** We searched PubMed, Ovid, and Web of Science databases for articles on studies using the ALFF/fALFF method in patients with aMCI and vMCI. Based on the activation likelihood estimation (ALE) method, connectivity modeling based on coordinate meta-analysis and functional meta-analysis was carried out.

**Results:** Compared with healthy controls (HCs), patients with aMCI showed increased ALFF/fALFF in the bilateral parahippocampal gyrus/hippocampus (PHG/HG), right amygdala, right cerebellum anterior lobe (CAL), left middle temporal gyrus (MTG), left cerebrum temporal lobe sub-gyral, left inferior temporal gyrus (ITG), and left cerebrum limbic lobe uncus. Meanwhile, decreased ALFF/fALFF values were also revealed in the bilateral precuneus (PCUN), bilateral cuneus (CUN), and bilateral posterior cingulate (PC) in patients with aMCI. Compared with HCs, patients with vMCI predominantly showed decreased ALFF/fALFF in the bilateral CUN, left PCUN, left PC, and right cingulate gyrus (CG).

**Conclusions:** The present findings suggest that ALFF and fALFF displayed remarkable altered patterns between aMCI and vMCI when compared with HCs. Thus, the findings of this study may serve as a reliable tool for distinguishing aMCI from vMCI, which may help understand the pathophysiological mechanisms of these diseases.

## Introduction

Mild cognitive impairment is a nosological entity referred to as a cognitive decline that is beyond normal peers. The condition is considered to be the transitional state between normal aging and dementia, where activities of daily living are unaffected (Sanford, 2017; Petersen et al., 2018). Amnestic mild cognitive impairment (aMCI) and vascular mild cognitive impairment (vMCI) are the two most common forms of the pre-dementia subtypes (Wentzel et al., 2001; Sun et al., 2016). The aMCI is characterized by isolated episodic memory impairment associated with higher risk; it is also considered as the prodromal state for the development of Alzheimer's disease (AD) (Yan et al., 2019). The vMCI, on the other hand, is described as an abnormal condition caused by vascular diseases, where the cognitive impairment of the patient is not serious and does not meet the criteria of dementia (Consoli et al., 2012). Interests in diagnosing and distinguishing between aMCI and vMCI have attracted a lot of attention and have brought out a great deal of research in both clinical and research settings. From this, researchers proposed that early diagnosis and active intervention could effectively delay the progression from MCI to dementia (Sanford, 2017). Currently, the clinical and research diagnostic criteria for aMCI and vMCI mainly depend on clinical history, neuropsychological assessment, and neuroimaging examination (Sudo et al., 2015; Anderson, 2019), but it is still difficult to distinguish between these two forms of cognitive impairment at an early stage. Therefore, a study on the similarities and specificities between aMCI and vMCI in MRI may provide a new prospect for the diagnosis and differentiation of these two diseases.

Resting-state functional MRI (rs-fMRI) has also been widely used to study the internal brain function of patients with many neuropsychiatric diseases, including MCI (Ni et al., 2016; Yang et al., 2018; Xu et al., 2020). The amplitude of low-frequency fluctuations (ALFF) of the rs-fMRI is a method to measure low-frequency oscillations of the blood-oxygen-level-dependent (BOLD) signal and local spontaneous activity during the resting state (Zang et al., 2007; Zou et al., 2008; Xi et al., 2013). Several studies have shown that ALFF can be used as an indicator of the disease state of the brain (Han et al., 2011; Chen et al., 2015), but it may be affected by a number of non-neurophysiological fluctuations. The fractional amplitude of low-frequency fluctuations (fALFF), on the other hand, represents the ratio of the amplitude in the low-frequency range to the sum of the amplitude in the whole frequency range (Wang et al., 2016). It has high sensitivity and specificity in the detection of spontaneous brain activity, but it is not as reliable as ALFF (Zou et al., 2008). These two indicators reflect the amplitude of low-frequency oscillations from different aspects and are sensitive indicators of related neurodegenerative changes (Wang et al., 2016). Both ALFF and fALFF have been more and more applied in numerous basic and clinical neuroscience studies with high reliability and reproducibility (Liu et al., 2017; Zhao et al., 2018; Luo et al., 2020). Moreover, ALFF and fALFF have been found to be abnormal in a number of neuropsychiatric disorders, such as AD, depression, and schizophrenia, and have also been found to be altered in aMCI and vMCI.

Previous ALFF/fALFF studies revealed abnormal intrinsic brain activity in aMCI. Xi et al. suggested that patients with aMCI, compared with healthy controls (HCs), showed decreased ALFFs in the left lateral temporal cortex, right hippocampus (Hip), parahippocampal gyrus (PHG), and right ventral medial prefrontal cortex (vMPFC), while increased ALFFs were displayed in the left temporal-parietal junction (TPJ) and inferior parietal lobule (IPL) (Xi et al., 2013). Meanwhile, a machine learning method demonstrated the gradual disturbances of the ALFF/fALFF in the AD spectrum as disease advanced. These studies showed several brain regions with decreased ALFF/fALFF within different bands among the bilateral cingulum, bilateral inferior cerebellum lobe, and bilateral precuneus (PCUN). However, increased ALFF/fALFF were also detected in the hip, frontal lobe, and paracentral lobe and involved in default-mode regions, such as the hip, PHG, posterior cingulate gyrus (PCG), and middle frontal gyrus (MFG). These abnormities were significantly correlated with the neuropsychological assessments as diseases progressed (Long et al., 2016; Yang et al., 2018). More recently, vascular risk factors have been found to modulate the spontaneous brain activity in patients with MCI, thus providing preliminary evidence that MCI patients with high vascular risk demonstrated decreased ALFF in the left Hip as compared with HCs with high vascular risk. This may serve as a potential neuroimaging biomarker for an underlying vascular contribution to AD (Zhuang et al., 2020). Previous studies have shown that ALFF/fALFF changes are closely related to cognitive function in patients with AD, MCI, white matter osteoporosis, and cerebral autosomal dominant arteriopathy with subcortical infarcts and leukoencephalopathy (CADASIL), suggesting that ALFF/fALFF may be an imaging biomarker for these diseases (Li et al., 2017; Yang et al., 2018; Su et al., 2019; Wang J. et al., 2019; Wang P. et al., 2019). Thus, the study of ALFF/fALFF changes in aMCI and vMCI can help us find their imaging diagnostic markers and understand their pathophysiological mechanisms.

Considering the above-mentioned ALFF and fALFF findings, in this study, we used the activation likelihood estimation (ALE) method to study ALFF/fALFF changes in aMCI and vMCI to explore their ALFF change pattern compared to HCs and diagnose and differentiate aMCI from vMCI at an early stage. Since there have been many reports of decreased ALFF/fALFF in the PCUN and posterior cingulate cortex (PCC) in aMCI and vMCI (Jing et al., 2012; Ding et al., 2015; Ni et al., 2016; Yang et al., 2018), we hypothesized that aMCI and vMCI also follow this pattern and are expected to find changes in ALFF/fALFF in some other brain regions, which may serve as a reliable neuroimaging biomarker for the two subtypes of MCI.

## Methods

### Literature Search and Selection Criteria

This study followed the list of the Preferred Reporting Items for Systematic Reviews and Meta-analyses (PRISMA) statement and the phase flowchart for meta-analysis (Liberati et al., 2009; Moher et al., 2009).

### Search Strategy

Studies were comprehensively searched in the PubMed, Web of Science, and Ovid databases. Search keywords were as follows: (“vascular cognitive impairment” OR “vascular cognitive impairment-no dementia” OR “vascular mild cognitive impairment” OR “amnestic mild cognitive impairment” OR “mild cognitive impairment”) AND (“amplitude of low-frequency fluctuations” OR “fractional amplitude of low-frequency fluctuations”). Considering that different articles may use different terms to describe vMCI, in order to ensure the comprehensiveness of the search, a supplementary search was made for vMCI. The search keywords are as follows: (“small vessel disease” OR “vascular cognitive impairment not dementia” OR “subcortical ischemic vascular disease” OR “moyamoya disease” OR “Leukoaraiosis” OR “leukodystrophy” OR “CADASIL” OR “vascular deficit” OR “vascular disorder” OR “cerebrovascular disorder” OR “cerebrovascular deficit” OR “vascular” OR “cerebrovascular”) AND (“amplitude of low-frequency fluctuations” OR “fractional amplitude of low-frequency fluctuations”). All articles published up to and including March 2021 were examined; thus, a total of 515 articles were studied.

### Inclusion and Exclusion Criteria

Criteria for inclusion were as follows: (1) The patients met the diagnostic criteria for aMCI or vMCI; (2) the patients were compared with HCs for ALFF/fALFF; (3) information on three-dimensional Talairach or Montreal Neurological Institute (MNI) coordinates was reported; (4) the study was based on rs-fMRI; and (5) the research was written in English and published in a peer-reviewed journal.

Criteria for exclusion were as follows: (1) The study was based on other diseases, such as schizophrenia and epilepsy; (2) the study was categorized as a case report or secondary literature (e.g., systematic review and meta-analysis).

### Data Extraction and Quality Assessment

The research results were screened independently by two authors (Xulian Zhang and Chen Xue) according to the inclusion and exclusion criteria. In case of disagreement, the reviewers (Xuan Cao and Qinlging Huang) evaluated and made the final decision. Firstly, we conducted a preliminary screening of the titles and abstracts of the studies to evaluate whether they conformed to the research content being explored. Secondly, for articles that conformed to the research content or with content that could not be determined according to the title and abstract, the full text was reviewed for a more extensive assessment. Thirdly, the articles obtained after preliminary screening were examined again to assess whether they met the inclusion criteria. Finally, we cross-checked the references of all the retrieval results to find the missing studies.

### Data Analysis Procedures

The results of that compared aMCI with HCs and vMCI with HCs were divided into three groups according to decreased or increased ALFF/fALFF values: aMCI increased ALFF/fALFF (*n* = 377; 43 foci); aMCI decreased ALFF/fALFF (*n* = 351; 61 foci); and vMCI decreased ALFF/fALFF (*n* = 136; 20 foci).

JAVA GingerALE Version 2.3.6 (http://www.brainmap.org/ale) was used for meta-analysis free of charge and for calculating the ALFF changes in amnestic and vMCI compared to HCs based on the method of ALE. First, the foci data recorded in the text file were imported into the reading software (Eickhoff et al., 2012), and coordinates in the Talairach space were converted into the MNI 152 standard space using the GingerALE converter foci tool. Then, the threshold for using the error discovery rate in the ALE map was set to *p* < 0.05 (Eickhoff et al., 2012). Finally, the ALE map was overlaid into the MNI 152 template and viewed using the DPABI software (http://rfmri.org/DPABI).

## Results

### Search Results

**Figure 1:**
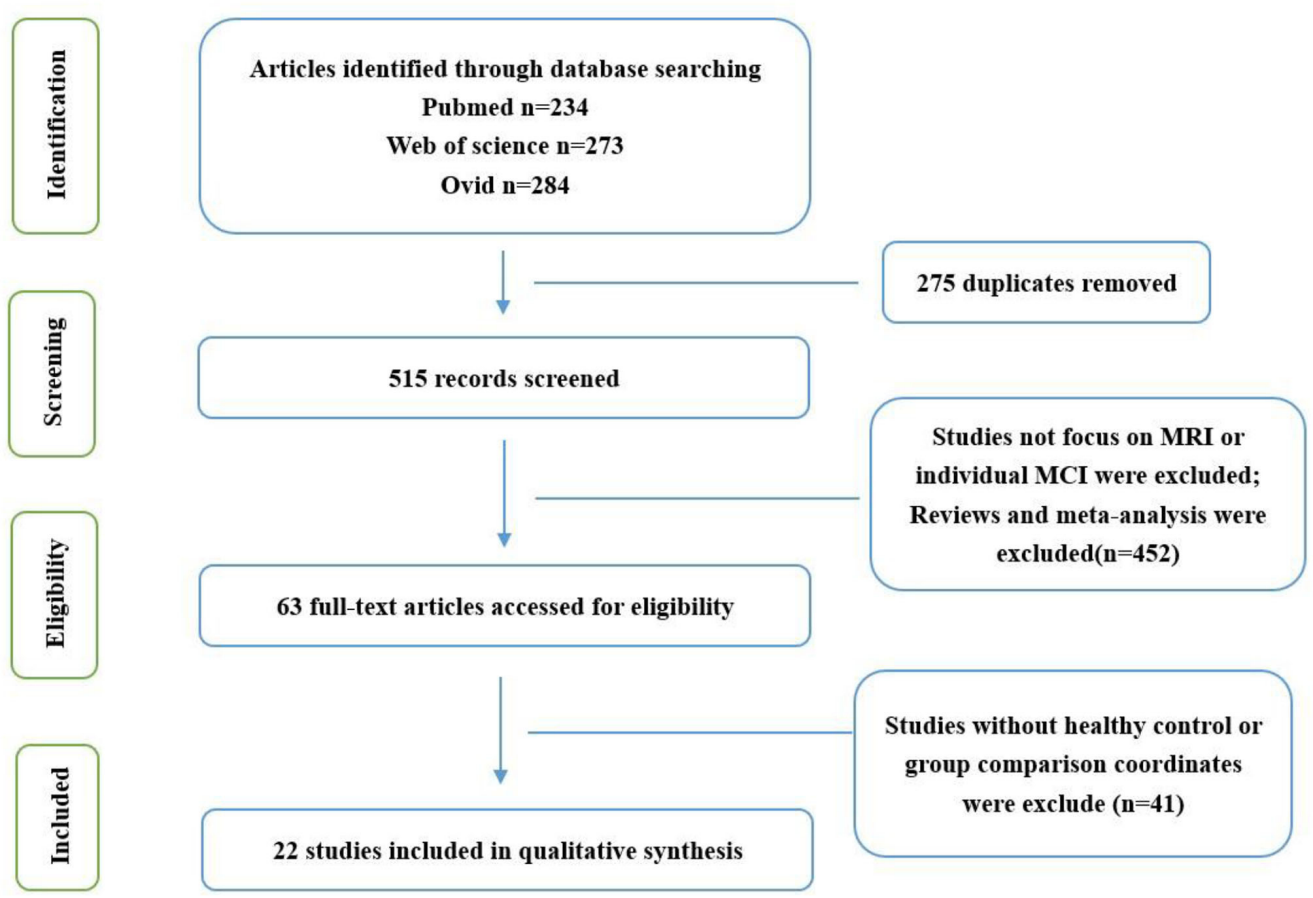
Flowchart to identify the studies that are eligible for systematic review.

After the preliminary screening of the retrieval results, 62 studies were obtained, of which 21 were excluded because they focused on other diseases or meta-analysis, 14 were excluded because they did not have an HC group or group comparison coordinates, and 5 were excluded because they were not published in English. Finally, 22 studies were included in the present meta-analysis (Figure 1; Table 1).

**Table 1:** Demographic characteristics of the included studies.

**Study**	**Imaging modality**	**N**	**Age (SD)**	**Gender (male/female)**	**MMSE (SD)**	**Group contrasts**	**Foci**
**ALFF/fALFF IN THE aMCI PATIENTS**							
**ALFF**							
Xi et al. (2013)	rs-fMRI	aMCI 18	67.39 (7.67)	8/10	25.16 (3.43)	MCI > HC	2
		HC 20	65.42 (5.75)	9/11	28.14 (1.84)	MCI < HC	3
Cai et al. (2017)	rs-fMRI	aMCI 39	72.4 (5.01)	19/20	25.52 (2.88)	MCI > HC	5
		HC 38	73.92 (3.90)	19/19	29.28 (0.88)	MCI < HC	7
Yin et al. (2014)	rs-fMRI	aMCI 11	66.6 (8.7)	2/9	24.6 (3.2)	MCI > HC	2
		HC 22	62.1 (8.1)	12/10	29.2 (1.1)	MCI < HC	4
Li et al. (2014)	rs-fMRI	aMCI 17	67.0 (7.9)	9/8	25.6 (1.27)	MCI > HC	5
		HC 22	62.6 (5.8)	11/11	28.5 (1.1)	MCI < HC	2
Ni et al. (2016)	rs-fMRI	aMCI 26	71 (9)	12/14	25 (1.48)	MCI > HC	2
		HC 28	70 (9)	17/11	29 (1.09)	MCI < HC	5
Wang et al. (2011)	rs-fMRI	aMCI 16	69.38 (7.00)	7/9	26.50 (1.03)	MCI > HC	0
		HC 22	66.55 (7.67)	7/15	28.59 (0.59)	MCI < HC	2
Zhuang et al. (2020)	rs-fMRI	aMCI 43	64.5 (5.64)	18/25	26.77 (1.66)	MCI > HC	2
		HC 29	66.79 (3.68)	7/22	28.71 (0.91)	MCI < HC	0
Liang et al. (2014)	rs-fMRI	aMCI 53	73.2 (7.3)	22/31	27.1 (2.3)	MCI > HC	1
		HC 35	74.3 (5.9)	17/18	28.9 (1.6)	MCI < HC	8
**fALFF**							
Yang et al. (2020)	rs-fMRI	aMCI 52	68.06 (9.32)	26/26	24.52 (4.27)	MCI > HC	2
		HC 55	63.41 (7.97)	22/23	28.07 (2.14)	MCI < HC	7
Liu et al. (2020)	rs-fMRI	aMCI 20	68.8 (11.2)	12/8	27.4 (1.66)	MCI > HC	4
		HC 22	72.7 (8.05)	9/13	28.3 (1.42)	MCI < HC	4
Zhou et al. (2020)	rs-fMRI	aMCI 24	69.8 (6.2)	10/14	23.9 (3.6)	MCI > HC	4
		HC 32	67.9 (6.4)	14/18	28.0 (1.9)	MCI < HC	0
Zhao et al. (2015)	rs-fMRI	aMCI 34	68.0 (7.6)	14/20	25.5 (1.6)	MCI > HC	0
		HC 34	66.9 (6.7)	18/16	29.2 (0.9)	MCI < HC	2
Jing et al. (2012)	rs-fMRI	aMCI 10	78.42 (9.65)	5/5	–	MCI > HC	2
		HC 8	75.35 (6.45)	3/5	–	MCI < HC	0
Li et al. (2017)	rs-fMRI	aMCI 27	67.44 (8.49)	13/14	23.52 (3.31)	MCI > HC	0
		HC 32	64.88 (7.54)	16/16	27.69 (1.67)	MCI < HC	9
Yang et al. (2018)	rs-fMRI	aMCI 55	67.51 (9.62)	27/28	24.66 (4.20)	MCI > HC	4
		HC 57	63.77 (8.09)	22/35	28.14 (2.13)	MCI < HC	8
**ALFF IN THE vMCI PATIENTS**							
Ni et al. (2016)	rs-fMRI	vMCI 22	79 (6)	16/6	25 (1.48)	MCI > HC	2
		HC 28	70 (9)	17/11	29 (1.09)	MCI < HC	2
Ding et al. (2015)	rs-fMRI	vMCI 11	63.09 (4.99)	6/5	–	MCI > HC	4
		HC 12	63.64 (5.35)	6/6	–	MCI < HC	4
Li et al. (2015)	rs-fMRI	vMCI 28	67.9 (6.1)	16/12	27.89 (1.57)	MCI > HC	3
		HC 30	66.6 (4.6)	14/16	28.10 (1.73)	MCI < HC	2
Wang J. et al. (2019)	rs-fMRI	vMCI 28	59.28 (6.12)	14/14	24.96 (1.48)	MCI > HC	1
		HC 28	58.35 (6.82)	13/15	29.46 (1.07)	MCI < HC	1
Su et al. (2019)	rs-fMRI	vMCI 22	49.0 (14.2)	13/9	23.3 (6.3)	MCI > HC	3
		HC 44	48.5 (13.7)	26/18	28.6 (1.1)	MCI < HC	1
Lei et al. (2014)	rs-fMRI	vMCI 11	40.2 (11.2)	4/7	19.6 (4.3)	MCI > HC	15
		HC 22	40.2 (7.2)	10/12	29.0 (1.2)	MCI < HC	3
Ding et al. (2018)	rs-fMRI	vMCI 14	67.9 (8.7)	8/6	26.87 (0.32)	MCI > HC	2
		HC 15	65.8 (7.9)	7/8	28.51 (0.28)	MCI < HC	7

### Meta-Analysis Results

#### Abnormal ALFF/fALFF in aMCI

**Figure 2:**
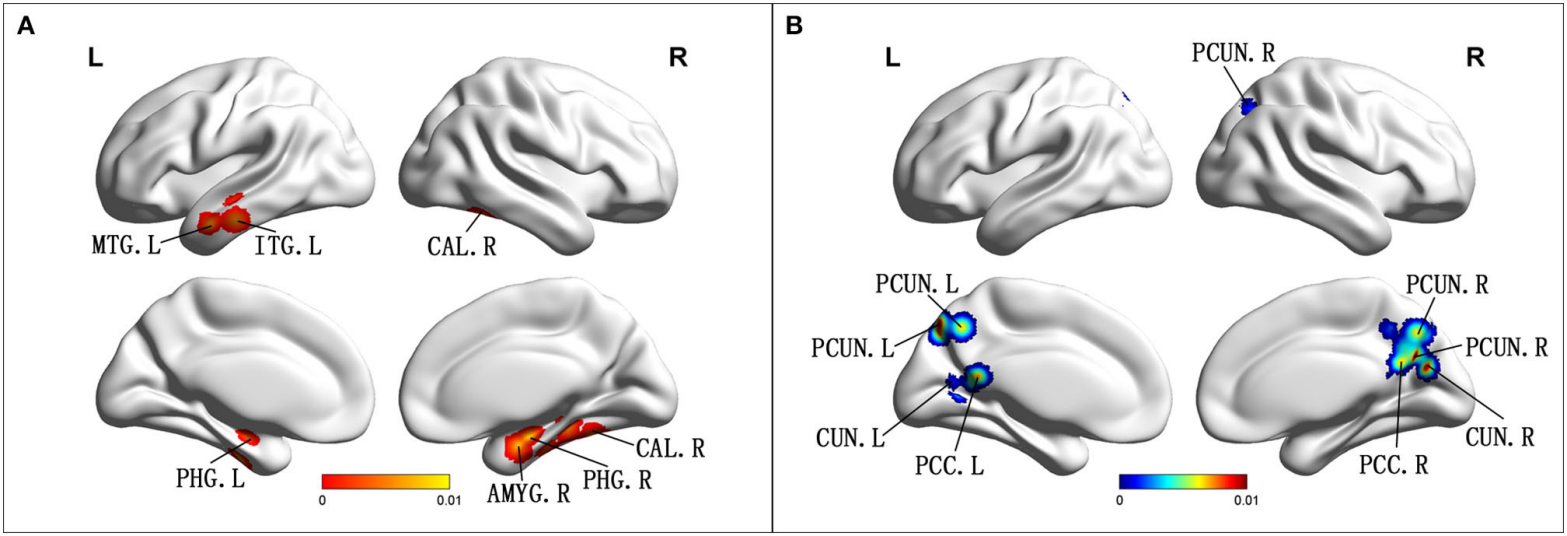
**(A)** Brain regions showing increased ALFF/fALFF in patients with aMCI compared with HCs. **(B)** Brain regions showing decreased ALFF/fALFF in patients with aMCI compared with HCs. aMCI, amnestic mild cognitive impairment; HCs, healthy controls; ALFF/fALFF, the amplitude of low-frequency fluctuation/fractional amplitude of low-frequency fluctuation; MTG, middle temporal gyrus; ITG, inferior temporal gyrus; CAL, cerebellum anterior lobe; PHG, parahippocampal gyrus; AMYG amygdala; PCUN, precuneus; CUN, cuneus; PCC, posterior cingulate; R, right; L, left.

Compared with HCs, patients with aMCI showed increased ALFF/fALFF in the bilateral PHG/Hip, right amygdala (AMYG), right cerebellum anterior lobe (CAL), left middle temporal gyrus (MTG), left cerebrum temporal lobe sub-gyral, left inferior temporal gyrus (ITG), and left cerebrum limbic lobe uncus (Table 2; Figure 2A). Patients with aMCI also showed decreased ALFF/fALFF in the bilateral PCUN, bilateral cuneus (CUN), and bilateral PCC (Table 2; Figure 2B).

**Table 2:** All clusters from the activation likelihood estimation (ALE) analysis.

**Cluster**	**Volume (mm^**3**^)**	**MNI**			**Anatomical regions**	**MaximumALE value**	**Side**	**BA**
		**X**	**Y**	**Z**				
**ALFF/fALFF IN THE aMCI PATIENTS**								
**MCI** **>** **HC**								
1	12120	36	−36	−16	Parahippocampal Gyrus	0.009105647	Right	36
1	12120	30	−6	−26	Amygdala	0.008697757	Right	–
1	12120	34	−12	−22	Parahippocampal Gyrus/Hippocampus	0.008655652	Right	–
1	12120	42	−24	−24	Parahippocampal Gyrus	0.008040754	Right	36
1	12120	38	−20	−24	Parahippocampal Gyrus/Hippocampus	0.007865525	Right	–
1	12120	36	−50	−18	Cerebellum Anterior Lobe	0.007794415	Right	–
2	11160	−36	−10	−22	Parahippocampal Gyrus/Hippocampus	0.009343305	Left	–
2	11160	−60	0	−24	Middle Temporal Gyrus	0.009123246	Left	21
2	11160	−50	−18	−16	Sub-Gyral	0.008646758	Left	21
2	11160	−62	−18	−24	Inferior Temporal Gyrus	0.008411912	Left	20
2	11160	−38	−14	−32	Uncus	0.007911957	Left	20
**MCI** **<** **HC**								
1	23032	−10	−60	10	Cuneus	0.014755126	Left	30
1	23032	12	−66	28	Precuneus	0.011124825	Right	31
1	23032	−10	−74	48	Precuneus	0.010285008	Left	7
1	23032	18	−68	42	Precuneus	0.010156754	Right	7
1	23032	10	−72	22	Cuneus	0.00971911	Right	18
1	23032	18	−54	24	Posterior Cingulate	0.009503377	Right	31
1	23032	−14	−72	42	Precuneus	0.009484317	Left	7
1	23032	4	−58	24	Posterior Cingulate	0.009442661	Right	23
1	23032	−4	−52	14	Posterior Cingulate	0.009429278	Left	29
1	23032	10	−66	42	Precuneus	0.008669218	Right	7
1	23032	−6	−60	44	Precuneus	0.006714426	Left	7
1	23032	14	−52	44	Precuneus	0.00636507	Right	7
**ALFF/fALFF IN THE vMCI PATIENTS**								
**MCI** **<** **HC**								
1	17944	6	−81	30	Cuneus	0.008325707	Right	18
1	17944	−8	−80	42	Cuneus	0.008055744	Left	19
1	17944	0	−66	54	Precuneus	0.006901417	Left	7
1	17944	−2	−52	66	Precuneus	0.006225559	Left	7
2	16000	−4	−46	24	Posterior Cingulate	0.01040697	Left	30
2	16000	−4	−54	20	Posterior Cingulate	0.007187156	Left	30
2	16000	8	−50	32	Cingulate Gyrus	0.006430536	Right	31

#### Abnormal ALFF/fALFF in vMCI

**Figure 3:**
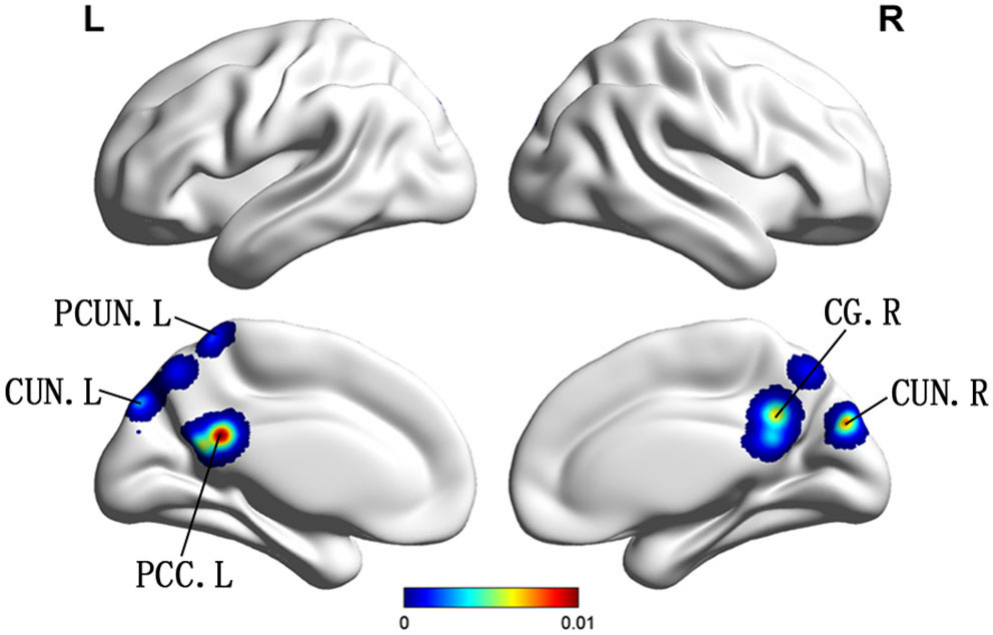
Brain regions showing decreased ALFF in patients with vMCI compared with HCs. PCUN, precuneus; CUN, cuneus; PCC, posterior cingulate; CG, cingulate gyrus R, right; L, left.

Compared with HC, patients with vMCI showed decreased ALFF/fALFF in the bilateral CUN, left PCUN, left PCC, and right cingulate gyrus (CG) (Table 2; Figure 3).

## Discussion

This study is the first meta-analysis to investigate the changes of ALFF/fFALFF in aMCI and vMCI and further explore whether these changes contribute to the diagnosis and differentiation of the two diseases. Compared with HCs, we found that the ALFF/fALFF values of both aMCI and vMCI were altered, which was consistent with the findings of the previous studies (Xi et al., 2013; Yin et al., 2014). In patients with aMCI, ALFF/fALFF increased mainly in the bilateral PHG/Hip, right AMYG, right CAL, left MTG, left cerebrum temporal lobe sub-gyral, left ITG, and left cerebrum limbic lobe uncus, while these values decreased mainly in the bilateral PCUN, bilateral CUN, and bilateral cingulate cortex. However, we only found that the ALFF/fALFF decreased in the left side of the bilateral CUN, the left PCC of the PCUN, and the right CG in patients with vMCI, but no brain regions with increased ALFF/fALFF values were found.

In this study, we found that, in aMCI compared to HCs, the brain regions with increased ALFF/fALFF were mainly concentrated in the limbic lobe, MTG, ITG, and anterior cerebellar lobe, while the brain regions with decreased ALFF/fALFF were mainly concentrated in the parietal lobe, occipital lobe, and limbic lobe. A quantitative meta-analysis found that patients with aMCI showed increased ALFF/fALFF in the right CAL, right PCUN, right IPL, and left ITG, while decreased ALFF/fALFF was found in the right PCUN and PCC. These results are mostly consistent with our findings (Xu et al., 2020). Meanwhile, increased ALFF/fALFF also occurred in the right AMYG and right CAL within our meta-data. A voxel-based morphometry meta-analysis found that the aMCI group showed significant GM atrophy in the left AMYG and right Hip, and these findings were highly consistent with the Alzheimer's Disease Neuroimaging Initiative (ADNI) dataset. These abnormalities further confirmed that GM atrophy is accompanied by local ALFF/fALFF abnormalities in patients with aMCI (Zhang J. et al., 2021). However, different results have been reported previously. Studies have revealed that aMCI groups showed increased ALFF in the calcarine, right cuneus, and bilateral PC/PCUN, and decreased ALFFs in the left inferior frontal gyrus, superior temporal gyrus, and insula (Liu et al., 2014; Zhuang et al., 2019). This may be due to the frequency bands chosen (Slow-4 and Slow-5). An rs-fMRI study showed abnormal ALFF/fALFF in the Slow-5 band of PCC/PCU and PHG, and that several occipital regions were greater than the Slow-4 band in patients with aMCI compared with age- and sex-matched HCs. These abnormalities reflect the functional differences between groups that rely on these frequency bands (Han et al., 2011). In addition, some studies have found that the PICALM rs541458 and TOMM40 gene polymorphisms can regulate ALFF in elderly patients with aMCI (Liu et al., 2014; Zhuang et al., 2019). From the above, we can see that changes in ALFF/fALFF are the result of the combined action of many factors. Although our results are broadly consistent with those of most previous studies (Xi et al., 2013; Yin et al., 2014), there are still some differences, which may be related to the influence of multiple factors on ALFF/fALFF changes.

The limbic lobe mainly includes the hip, parahippocampal gyrus, CG, and AMYG and is mainly involved in emotion and motivation functions (Heimer and Van Hoesen, 2006). Studies have shown that the hip and its parahippocampal gyrus play an important role in memory function, which is mainly related to information storage and episodic memory retrieval (Xi et al., 2013). Heimer et al. found that the hip and CG played a role in regulating emotional state and the AMYG was mainly involved in the recognition of emotional meaning and the generation of emotional state (Heimer and Van Hoesen, 2006). A meta-analysis by Davey et al. found that the MTG is an important junction between the default mode network (DMN) and the multi-need network and is mainly involved in semantic control, while the ITG is mainly involved in higher cognitive functions such as language and vision (Davey et al., 2016; Lin et al., 2020). The anterior cerebellum is known to be involved in sensorimotor activity, but studies have also suggested that it plays an important role in cognition and emotion (Schmahmann, 2019). In addition, previous studies have found that the PCUN/CUN is structurally and functionally connected to the DMN, which may play a central role in the neural network related to consciousness (Cavanna, 2007; Cunningham et al., 2017; Su et al., 2019). Although the conclusions of studies on ALFF have exhibited some inconsistency, a meta-analysis of rs-fMRI studies using the seed-based mapping software package revealed widespread aberrant regional spontaneous brain activity in aMCI and a regression analysis found that the severity of cognitive impairment in aMCI was negatively correlated with increased ALFFs in the CUN/PCUN cortices. These results were consistent with our meta-analysis results (Pan et al., 2017). We also found a meta-analysis based on brain 18F-fluorodeoxyglucose positron emission tomography (FDG-PET), which found that the left PCC/PCUN was the most robust and reliable metabolic altered brain region for metabolic alterations in aMCI converted to AD. The hypometabolism in the left PCC/PCNU and altered fMRI may serve as a potential biomarker for AD and other forms of cognitive impairment (Ma et al., 2018; Zhang Q. et al., 2021). These findings support our meta-analysis results that found that these aberrant regions may be regarded as early neuroimaging biomarkers for aMCI (Lau et al., 2016).

Our study found that, compared with HCs, vMCI showed no significant difference in ALFF/fALFF increased brain regions, while the decreased brain regions were the bilateral CUN, left PCUN, left PCC, and right CG. These brain areas are essentially the same as those that were decreased in patients with aMCI. Previous researchers have also found consistent results based on rs-fMRI in vMCI. A study on leukoaraiosis (LA) divided LA patients into two groups of LA-vMCI and LA with vascular dementia (LA-VaD). The ANOVA statistical analysis showed the predominant and widespread differences of ALFF in the PCC/PCUN and the right ITG for LA patients compared with the HCs. In particular, ALFF was found to be significantly decreased in the PCC/PCUN and increased in the temporal regions for LA-VaD patients, while the LA-vMCI group showed significantly increased ALFFs in the ITG compared to the HCs and the LA-VaD group. Furthermore, the results revealed that decreased executive functions were correlated with altered ALFF in the left PCUN (Wang J. et al., 2019). In addition, studies have shown that CG is related to cognitive processes and behaviors, which may be the reason why vMCI is mainly shown as decreased processing speed and executive ability (Vasquez and Zakzanis, 2015; Apps et al., 2016). In view of our meta-analysis results between aMCI and vMCI groups, we found that decreased ALFF/fALFF in the PCC/PCUN and CG both occurred in the two groups. Our results are consistent with the aforementioned meta-analysis, which may indicate that there may be some similarity in cognitive impairment caused by different brain etiologies.

However, decreased ALFF/fALFF in the PCUN/CUN and CG may present a decompensated stage of cognitive impairment in aMCI and vMCI, and this may contribute to the understanding of the pathophysiology and interconnectivity of disparate cognitive processes. Patients with aMCI not only displayed decreased ALFF/fALFFs in several different brain regions but also demonstrated increased ALFF/fALFFs in other brain regions. Meanwhile, we could not achieve satisfactory results regarding the increased ALFF/fALFF in vMCI even after we increased the statistical threshold. Through careful observation, we found that all the included studies on vMCI had brain regions with increased ALFF/fALFF, but there were different opinions on the specific brain regions with increased ALFF/fALFF. We speculated that the inclusion criteria of vMCI and the frequency bands selected in the studies might be related. This may explain the absence of elevated brain regions found in patients with vMCI. Although ALFF/fALFF abnormalities may depend on different frequency bands, these increased ALFF/fALFF in patients with aMCI may still indicate a compensatory mechanism in the early stage of cognitive impairment.

## Limitations

The limitations of this meta-analysis mainly include the following points. Firstly, different studies in the included literature used different criteria to distinguish vMCI from HCs; thus, there is no strict unified standard yet. This may be because the concept of vMCI was only proposed in recent years, and previous studies had inconsistent statements on the disease. Secondly, our meta-analysis was based on a whole-brain analysis, without specific analysis of the various networks in the brain. Finally, although some brain regions of aMCI and vMCI were found to be different from those of HCs, we did not have enough evidence to indicate which brain regions that ALFF/fALFF changes are the early neuroimaging biomarkers of aMCI and vMCI. But finding them is going to be an important part of our future work.

## Conclusions

This study showed that aMCI and vMCI had different ALFF/fALFF changes compared with HCs. Taken together, our findings provide novel insights into the pathophysiological mechanisms of aMCI and vMCI and may be helpful to distinguish aMCI from vMCI for early clinical interventions.

## Data Availability Statement

The raw data supporting the conclusions of this article will be made available by the authors, without undue reservation.

## Author Contributions

XZ, CX, and QH: designed the study. XZ, CX, QH, XC, QY, WQ, WX, and SZ: collected the data. XZ and CX: analyzed the data and prepared the manuscript.

## Conflict of Interest

The authors declare that the research was conducted in the absence of any commercial or financial relationships that could be construed as a potential conflict of interest.

## Publisher's Note

All claims expressed in this article are solely those of the authors and do not necessarily represent those of their affiliated organizations, or those of the publisher, the editors and the reviewers. Any product that may be evaluated in this article, or claim that may be made by its manufacturer, is not guaranteed or endorsed by the publisher.
